# Transfer of DNA from Bacteria to Eukaryotes

**DOI:** 10.1128/mBio.00863-16

**Published:** 2016-07-12

**Authors:** Benoît Lacroix, Vitaly Citovsky

**Affiliations:** Department of Biochemistry and Cell Biology, State University of New York, Stony Brook, New York, USA

## Abstract

Historically, the members of the *Agrobacterium* genus have been considered the only bacterial species naturally able to transfer and integrate DNA into the genomes of their eukaryotic hosts. Yet, increasing evidence suggests that this ability to genetically transform eukaryotic host cells might be more widespread in the bacterial world. Indeed, analyses of accumulating genomic data reveal cases of horizontal gene transfer from bacteria to eukaryotes and suggest that it represents a significant force in adaptive evolution of eukaryotic species. Specifically, recent reports indicate that bacteria other than *Agrobacterium*, such as *Bartonella henselae* (a zoonotic pathogen), *Rhizobium etli* (a plant-symbiotic bacterium related to *Agrobacterium*), or even *Escherichia coli*, have the ability to genetically transform their host cells under laboratory conditions. This DNA transfer relies on type IV secretion systems (T4SSs), the molecular machines that transport macromolecules during conjugative plasmid transfer and also during transport of proteins and/or DNA to the eukaryotic recipient cells. In this review article, we explore the extent of possible transfer of genetic information from bacteria to eukaryotic cells as well as the evolutionary implications and potential applications of this transfer.

## INTRODUCTION

Vertical gene inheritance is the main pathway of transmission of genomic information from the parents to their offspring via germline or cell division. However, genetic information can be transmitted also between organisms that are not directly related; these exchanges are termed horizontal gene transfer (HGT; also known as lateral gene transfer) (1). Among prokaryotes, HGT—first observed as the spreading of drug resistance within a bacterial population (2)—is now recognized as a major evolutionary force (3–5). Indeed, several genome-wide studies have shown that HGT occurs at a high frequency between prokaryotic species, particularly if they are closely related or if they coexist in the same habitat or community, which provides many opportunities for DNA transfer (4, 5). Unlike evolution via gene duplications and mutations, a slow and incremental process, HGT permits fast acquisition of a new function important for species adaptation and survival.

Numerous cases of HGT from bacteria to eukaryotes have been demonstrated, although this process is assumed to be much less frequent than HGT between bacteria. The early evolution of eukaryotes was marked by endosymbiotic events leading to permanent acquisition of major organelles, e.g., mitochondria that originated from proteobacteria and plastids that originated from cyanobacteria, followed by organelle-to-nucleus gene transfer, usually referred to as endosymbiotic gene transfer (EGT) (6). Whereas the episodic gene transfer via EGT has a demonstrated evolutionary significance, the importance of HGT in the evolution of eukaryotes is still debated (7). A recent study analyzing a large number of protein sequences from bacterial and eukaryotic organisms indicates that gene inheritance in eukaryotes is predominantly vertical and suggests that HGT occurs only occasionally and that sequences acquired by HGT do not accumulate in eukaryotic genomes and do not contribute to long-term evolution of gene content (8). However, it is generally agreed that HGT from prokaryotes to eukaryotes does occur to a certain extent and, in some cases, plays a role in adaptive evolution (9). Whereas the identification of HGT genomic signatures indicates the existence of such events in the course of evolution, it does not inform about the pathway(s) and mechanisms(s) by which these sequences have been transferred. Instead, this information derives from numerous studies of known systems of natural and experimental gene transfer from bacteria to eukaryotic cells—such as the *Agrobacterium*-host plant interaction, the best-studied and best-understood system of transkingdom DNA transfer. Here, we review the major known cases of HGT from bacteria to eukaryotes that do not originate from prokaryote-derived permanent organelles, with a focus on natural and artificial prokaryote-to-eukaryote gene transfer systems that may help us understand the potential mechanisms involved in these transkingdom exchanges of genetic information.

## SIGNATURES OF BACTERIUM-TO-EUKARYOTE HGT IN GENOME SEQUENCES

In most cases, the first step in identifying an HGT event is the detection of a sequence that does not follow the expected phyletic distribution. However, the presence of such a sequence may also result from differential gene loss in most species of a single clade, leading to the impression that this gene is present only in one remaining species. The classical method to validate a suspected HGT event is phylogenetic inference: the finding of a topological disagreement between a strongly supported gene tree and the known species tree is a good indicator of an HGT event (4, 7, 10). Other accessory methods may help to confirm the occurrence of HGT, such as base composition, the presence or absence of introns, codon usage, synteny analysis, and such ecological features as a shared niche or location for the species involved. Still, the exact identity of the prokaryotic species from which the sequence acquired by HGT originates is sometimes difficult to determine because of subsequent evolution of the transferred sequence or because the “donor” species is extinct.

The complete sequencing of *Dictyostelium discoideum* (11) revealed 18 genes resulting from potential HGT from bacteria, which sometimes conferred new functions, such as a dipeptidase enzymatic activity potentially able to degrade the bacterial cell wall. Similarly, *Galdieria sulphuraria*, a red alga that lives in extreme, i.e., hot, acidic, and heavy-metal-rich, environments harbors genes obtained through HGT from bacteria and archaea and that may represent, after duplication and diversification, as much as 5% of its protein-encoding genes. Most of these proteins of suspected bacterial origin, such as an arsenic membrane protein pump similar to those found in thermoacidophilic bacteria, are expressed and are believed to have facilitated ecological adaptation of *G. sulphuraria* to extreme environments in the course of the evolution of this species (12).

Several cases of potential HGT from bacterial sources also have been identified in *Saccharomyces cerevisiae* and other yeast species (13, 14). For example, the *URA1* gene, which encodes the enzyme dihydroorotate dehydrogenase required for anaerobic synthesis of uracil, appears to originate from the lactic acid bacteria *Lactobacillales*. In plant-associated fungi, the acquisition of bacterial genes by HGT is considered widespread and likely represents a significant force in their adaptive evolution (15). These genes often encode factors involved in niche specification, pathogenicity, and adaptation to different metabolic requirements (10). Frequently, HGT occurs into plant-pathogenic fungi living in the community with many other plant-associated prokaryotic and eukaryotic microorganisms (16). In particular, within the genomes of three species of *Colletotrichum*, a genus of plant-pathogenic fungi that cause the crop-destructive disease anthracnose, at least 11 independent HGT events from bacterial genomes were identified (17). These transferred genes encode factors involved either in interaction with host plant and fungal virulence or in various metabolic processes, and they likely play a role for niche adaptation. Similarly, two species of the vascular wilt fungus *Verticillium* acquired by HGT from proteobacteria a gene that encodes a glucan glycosyltransferase involved in synthesis of extracellular glucans important for virulence (18).

Microbial eukaryotes, such as *D. discoideum* or *G. sulphuraria*, and most fungi either are unicellular or display a predominant unicellular stage in their life cycle; thus, in these species, genes can be vertically transmitted during cell division. However, for more complex, multicellular organisms, HGT can be transmitted vertically only by two general mechanisms: when the recipient cells are germline cells or when they are able to dedifferentiate and/or regenerate to a functional organism by asexual reproduction.

In multicellular organisms, bacterium-to-animal cell HGT appears to be limited to invertebrates, and it has originated either from gene transfer from endosymbiotic bacteria to their hosts or from transfer from bacteria to asexual animals (19). One of the most striking examples of HGT from bacterial endosymbionts to animal hosts is the gene transfer from *Wolbachia* to different arthropods and nematodes (20). *Wolbachia* species are good candidates for heritable HGT: they are intracellular symbionts, maternally inherited, and transmitted through egg cytoplasm. Among 11 sequenced arthropod genomes, eight contain *Wolbachia* sequences acquired via HGT; interestingly, the transferred sequences sometimes represented a significant portion (up to 30%) of the genome. HGT also had occurred from nonendosymbiont bacteria to freshwater asexual animals, such as *Hydra magnipapillata* (21) and bdelloid rotifers (22). On a few occasions, however, initial indications that animal genomes contain numerous genes originating from HGT were refuted by subsequent more-detailed analyses. For example, a large number of potential HGT events were first reported for the human genome (23), but this claim was disproved after closer examination of the data based on phylogenetic analysis that included a larger number of eukaryotic species (24).

A comparative genomic study of the early land plant *Physcomitrella patens* and of a flowering plant, *Arabidopsis*, uncovered 57 families of nuclear genes that potentially had been acquired by HGT, mainly from bacterial species (25). Several of these genes are involved in land-plant-specific activities, e.g., xylem formation, defense, and regulation of growth, suggesting that HGT played an important role in the transition from the aquatic to the terrestrial environment. Furthermore, phylogenetic evidence supports the idea that the major biosynthetic pathway of auxin, the main hormone of land plants, is derived from the bacterium-to-plant HGT (26). Another series of HGT events in plants resulted from the insertion of *Agrobacterium* transfer DNA (T-DNA) into the plant genome and its vertical transfer via sexual reproduction (27). It was first reported in *Nicotiana glauca* (28, 29) and then found in most species of *Nicotiana* tested to date (30, 31). In a screen of more than 100 dicotyledonous plant species, *Agrobacterium* T-DNA sequences were detected in two species of the genus *Linaria* (32). More recently, the presence of T-DNA sequences acquired by HGT was discovered in the genomes of several varieties of cultivated sweet potato, *Ipomoea batatas* (33). The origin of the T-DNA-derived genes was identified as a mikimopine strain of *Agrobacterium rhizogenes* for both *Nicotiana* and *Linaria* and likely as an ancestral form of *A. rhizogenes* for *I. batatas*. Some of these T-DNA genes are still expressed at a detectable level in modern plants, although whether they have a functional role in the plant biology remains unknown. These HGT events originating from *Agrobacterium* species represent a rare case for which the source of the transferred genes is clearly identified and the transfer pathway is well studied (see below).

## NATURAL AND EXPERIMENTAL BACTERIUM-TO-EUKARYOTE DNA TRANSFER SYSTEMS

**FIG 1:**
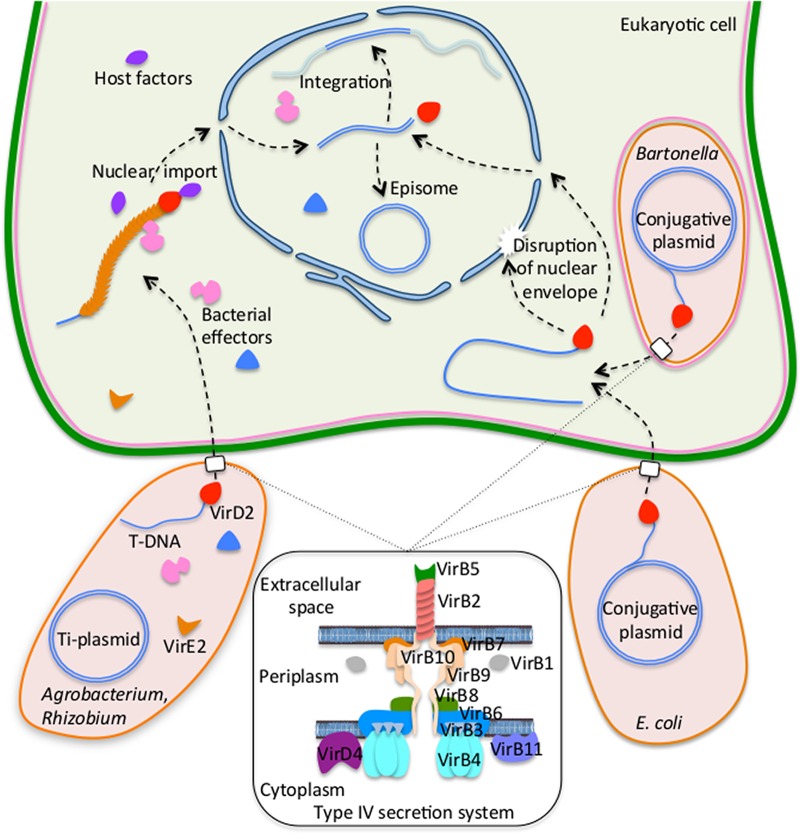
Schematic summary of known natural and experimental pathways for DNA transfer from bacteria to eukaryotic cells. *Agrobacterium* and related bacteria, *E. coli*, and *Bartonella henselae* can transfer DNA to different types of eukaryotic cells via the activity of their type IV secretion systems composed of VirD4/VirB proteins. Inside the host eukaryotic cell, the bacterial transferred DNA, usually a single-stranded molecule packaged into a nucleoprotein complex, is imported into the host nucleus. Nuclear import and further DNA processing, i.e., conversion to a double-stranded form, integration into the recipient cell genome, or formation of an episome, depend on interactions of the transferred DNA and its associated proteins with numerous host cell factors that represent different types of cellular machineries, such as nuclear import machinery, the ubiquitin/proteasome system, and DNA repair machinery. For further details, see the text.

The major known natural and artificial systems for gene transfer from bacterial to eukaryotic cells include such bacteria as *Agrobacterium* and *Rhizobium* species and *Escherichia coli*, and they are summarized in Fig. 1. The first system is the *Agrobacterium*-to-plant cell DNA transfer, which represents the paradigm of eukaryotic genetic transformation by bacteria and has long been considered a unique case in living nature; thus, it is the most-studied example of transkingdom gene transfer (34, 35). *Agrobacterium* is a plant-pathogenic bacterium that causes neoplastic growths, i.e., uncontrolled cell divisions that form galls or root proliferations, in its host plants by transferring a segment of DNA into the host cell genome. Most of the bacterial genes essential for gene transfer are located on a large tumor-inducing plasmid, termed the Ti plasmid, which also contains the transferred DNA segment, termed the T-DNA, that is delimited and specified by two short direct repeat sequences, left and right borders. Plant-derived phenolic and sugar signal molecules trigger the expression of the virulence (*vir*) genes in *Agrobacterium* cells, and the encoded Vir proteins mediate the transfer of the T-DNA to the plant cell. The T-DNA is transferred as a single-stranded molecule, produced by the VirD2 endonuclease, which, in association with the VirD1 DNA topoisomerase (36), mediates the mobilization of the transferrable T-DNA from the Ti plasmid by a strand replacement mechanism; VirD2 then remains covalently bound to the 5′ terminus of the T-DNA molecule. Upon interaction with the coupling factor VirD4, the VirD2–T-DNA complex is directed to the type IV secretion system (T4SS) composed of 11 proteins encoded by the *virB* operon. The T4SS then mediates the translocation of the VirD2–T-DNA complex, as well as several other Vir protein effectors, from the bacterium to the host cell cytoplasm. The fate of the T-DNA in the host cell relies on multiple interactions with *Agrobacterium* and host cell proteins, taking advantage of several host cell pathways to ensure the T-DNA nuclear import and integration into the host genome. Both VirD2 and the single-stranded DNA binding protein VirE2—which packages T-DNA into a helical nucleoprotein complex, termed the transfer (T) complex—can interact, directly or indirectly, with host factors to allow nuclear import of the T complex. This process likely occurs in a polar manner such that VirD2 directs the T-DNA to the nuclear pore while VirE2 facilitates the passage of the entire T complex through the pore via the importin-α-dependent nuclear import pathway. Inside the nucleus, the T complex is proteolytically uncoated from its associated bacterial and host proteins, presumably by interacting with the host ubiquitin/proteasome system (UPS). Then, the single-stranded T-DNA most likely is converted to a double-stranded form and integrated into the plant genome by the host DNA repair machinery. Interestingly, under laboratory conditions, *Agrobacterium* is able to transfer DNA to many nonplant cells, from fungi to human cultured cells (37), suggesting that T-DNA nuclear import and subsequent processing and integration are mediated by factors found in all diverse eukaryotic species, rather than by factors specific for host plants. Furthermore, *Agrobacterium* is also able to transfer sequences from a mobilizable plasmid (RSF1010) to plant cells via the activity of Vir proteins and of the plasmid mobilization functions (38).

As early as 1977, it was reported that the Ti plasmid of *Agrobacterium* could be transferred by conjugation to cells of the related species *Rhizobium trifolii*, which conferred on those bacteria the ability to trigger virulence, i.e., development of crown galls, on several plant species (39). Later, it was shown that the introduction of both a disarmed Ti plasmid, i.e., harboring the *vir* genes but no T-DNA, and a binary plasmid, i.e., containing T-DNA but no *vir* genes, into such *Rhizobiaceae* species as *Rhizobium leguminosarum*, *R. trifolii*, and *Phyllobacterium myrsinacearum*, produced virulent bacteria able to transfer T-DNA to *Arabidopsis*, tobacco, and rice (40), whereas potato plants were genetically transformed by *Sinorhizobium meliloti*, *Rhizobium* sp. strain NGR234, and *Mesorhizobium loti* supplied with a similar binary vector set (41). Another bacterial species of the *Rhizobiaceae* family, *Ensifer adherens*, when equipped with a cointegrated vector containing both the *vir* region and T-DNA from *Agrobacterium*, was used to transform potato and rice plants (42, 43). All these species are symbiotic bacteria that belong to the same *Rhizobiales* order as *Agrobacterium* and are able to mediate nitrogen assimilation for their host plants.

In all these studies, the *vir* region of a virulent strain of *Agrobacterium* had to be supplied along with the T-DNA in order to confer competence for plant transformation on the bacterial species, which naturally encoded no native plant transformation machinery. However, several species of rhizobia contain their own *vir* genes, with different levels of homology to the *Agrobacterium* vir genes. Specifically, *Rhizobium etli* strain CFN42 naturally contains, on its p42a plasmid, a functional T-DNA transfer machinery comprising all the necessary *vir* gene functions. Indeed, when a binary plasmid, containing a T-DNA but not the *vir* region, was introduced into *R. etli*, the resulting strain was able to transfer and integrate T-DNA into plant cells, albeit with a lower frequency than that with *Agrobacterium* (44). This T-DNA transfer was not observed with mutants of *R. etli* that lack one of the essential *vir* genes (*virG* or *virE2*) or with *R. leguminosarum*, a species very close to *R. etli* overall but with only very weak homology to the *vir* genes. Importantly, our analysis of the known DNA sequences of *R. etli* detected no homologies to known *Agrobacterium* T-DNA sequences, i.e., T-DNA borders or T-DNA genes; however, we cannot rule out the possibility that *R. etli* might contain its own, specific T-DNA sequences undetectable by *in silico* analyses.

The transfer of plasmid DNA can also occur from bacteria (*E. coli*) to yeast (*S. cerevisiae*) (45). This transfer was effective with both broad-host-range and F-factor plasmids, which represent the two main types of conjugative plasmids in Gram-negative bacteria. The transfer mechanism exhibited similarities with bacterial conjugation: for example, physical contact between the cells was required as well as genetic factors, i.e., *mob* and *oriT*, necessary for bacterial conjugation. DNA transfer was also observed from *E. coli* to other yeast species, such as *Kluyveromyces lactis* and *Pichia angusta* (46) or *Saccharomyces kluyveri* (47). The ability of *E. coli* to deliver DNA molecules via conjugation-like pathways to other types of eukaryotic cells was also reported for gene transfer to cultured human cells (48) and, more recently, to the unicellular algae diatoms (49). Although there is no definitive proof that such bacterium-to-eukaryote conjugation occurs in nature, these examples suggest that the ability to transfer genetic information to eukaryotic hosts is not restricted to *Agrobacterium*.

In another example of potential ability to genetically transform the host cell, *Bartonella henselae*, a facultative intracellular human bacterial pathogen, was shown to transfer a modified cryptic plasmid (50) or derivatives of the R388 plasmid (51) into human cultured cells via a conjugation-like mechanism. Indeed, the bacterial strains used in these experiments harbored a T4SS, which was required for the transport of plasmids from the bacterium to the host cell, and this DNA transfer was disrupted in *B. henselae* strains with a mutated *virB* region. Interestingly, host cell division was required for expression of the transgene, suggesting that the bacterium is unable to utilize the host nuclear import pathways and, instead, relies on breakdown of the host nuclear envelope. This *B. henselae*-human cell DNA transfer produced stable transgenic cell lines, indicating the integration of the transferred sequences in the host cell genome. Whereas the *Bartonella* T4SS is known to transport effector proteins essential for virulence into the host cells (52), its apparent ability to transfer plasmid DNA has no demonstrated role in the infection process and may represent the relic of an ancestral function.

## POTENTIAL MECHANISMS OF BACTERIUM-TO-EUKARYOTE HGT

DNA transfer into bacterial cells is known to occur via three different mechanisms: transformation (uptake of free DNA in solution), bacteriophage-mediated transduction (i.e., both generalized and specialized transduction), and plasmid-mediated transfer (i.e., conjugation, which usually requires close contact between donor and recipient cells). But are these mechanisms applicable to HGT? Potential HGT pathways may be inferred from studies of natural and experimental bacterium-to eukaryote gene transfer systems. Although yeast cells have been suggested to acquire exogenous DNA under conditions close to their natural environment (53), there are no known naturally occurring mechanisms of DNA uptake in eukaryotic cells. In a study of HGT between *Wolbachia* and *Aedes aegypti* (54), bacteriophage sequences were found close to the transferred genes, suggesting the role of bacteriophages as HGT vectors. It has also been suggested that some viruses, in particular giant viruses, may mediate transfer of DNA from bacteria to eukaryotes, but this pathway of HGT has not been confirmed experimentally (12).

Notably, most bacteria possessing the ability to transfer DNA to eukaryotic host cells belong to the *Alphaproteobacteria* class (55). Most of these bacterial species show a degree of interaction with eukaryotic hosts, from pathogenic or symbiotic lifestyles, e.g., *Agrobacterium* and *Rhizobium*, to optional or obligate parasitism of intracellular bacteria, e.g., *Bartonella* and *Wolbachia*. In these species, the “mobilome,” i.e., the pool of plasmids containing shared genetic information, plays a prominent role, which most likely underlies high genome plasticity and gene mobility between bacteria as well as the ability of these bacteria to transfer DNA to eukaryotic cells.

In known natural and experimental bacterium-to-eukaryote DNA transfer systems, i.e., bacterium-yeast conjugation, *Bartonella*-mediated transformation of animal cells, and *Agrobacterium*/*Rhizobium*-mediated genetic transformation of diverse eukaryotes, the transport of DNA from the bacterial cell to the recipient cell cytoplasm depends on conjugation-like mechanisms mediated by the bacterial T4SS. T4SSs are specialized molecular superstructures able to transport protein and DNA molecules between donor bacteria and a variety of recipient cells (56, 57). They are encoded by many bacterial species and are often involved in conjugation, i.e., transfer of genetic information between bacterial cells of the same or closely related species. However, T4SSs are also known to mediate macromolecular transport of DNA and/or proteins from bacteria to cells of their eukaryotic hosts. In fact, to date, T4SS represents the only demonstrated mechanism of transfer of genetic material to eukaryotic cells from bacteria in nature or under laboratory conditions. Although the export of macromolecules across the bacterial membranes and cell walls through the T4SS is well understood (57, 58), how the transported protein or DNA molecule passes across the eukaryotic recipient cell wall and membrane remains obscure. By analogy to type III secretion systems (T3SSs) (59), the T4SS pilus itself might pierce the host cell barriers to inject macromolecules directly into the host cytoplasm, but this mechanism has never been directly observed. The diameter of this channel is compatible with the size of transported macromolecules (60), and two studies have shown that DNA can be transferred by conjugation between bacterial cells without direct cell-to-cell contact, suggesting that DNA transits through the F pilus lumen (61, 62). Alternatively, the exported macromolecules first may be deposited at the surface of the recipient cell by the T4SS and then internalized by a separate mechanism that may involve host receptors and/or endocytosis. Indeed, studies of direct transformation of yeast cells have shown that once DNA molecules are placed at the surface of the host cell membrane, they may become internalized (e.g., reference 63).

Our knowledge about the molecular reactions that occur after the entry of the transferred DNA into the host cell cytoplasm and lead to the transgene expression and integration derives largely from the studies of *Agrobacterium*-mediated genetic transformation, which indicate that they rely on interactions of the transferred bacterial DNA and proteins with host factors. Specifically, in a eukaryotic cell, the incoming single-stranded DNA molecule, which represents a mobile T-DNA or a conjugative plasmid, should be imported into the cell nucleus before further processing. Nuclear uptake of the *Agrobacterium* T-DNA and its associated proteins is mediated by the host nuclear import machinery (35). In addition, it was suggested that, in some cases, the host cell division could be required for expression or integration of the invading DNA, thereby circumventing the necessity for active nuclear import and allowing passive entry in the nucleus following disruption of the nuclear envelope (50). Inside the nucleus, replication of the DNA, imported as a single-stranded molecule via the bacterial T4SS, is thought to occur before plasmid circularization or before integration (64–66) and is likely mediated by the host DNA replication machinery. In the case of *Agrobacterium*, the T-DNA circularization has been shown to occur, although it remains unclear whether these circles function as intermediates of integration (64–66). Furthermore, the single-stranded T-DNA molecule is converted to a double-stranded molecule before integration (67, 68), whereas the existence of other pathways of integration cannot be excluded. Finally, the host double-strand break (DSB) DNA repair pathways play a crucial role in integration of the transferred sequences (69).

## EVOLUTIONARY IMPLICATIONS OF HGT

A few decades ago, the only demonstrated bacterium-to-eukaryote gene transfer was represented by plant genetic transformation mediated by *Agrobacterium*. Subsequent studies showed that this capability could be observed in other related bacterial species, such as many *Rhizobiaceae*, including *R. etli*, *E. coli*, and *Bartonella*, and with a wider range of host species from all eukaryotic taxa. In addition, the ever-increasing amount of genomic data from both prokaryotes and eukaryotes led to the discovery of HGT genomic signatures in a broad spectrum of eukaryote species. Globally, the influence of bacterium-to-eukaryote HGT may have been more important for evolution of eukaryotes than previously thought, to the extent that HGT has been proposed to underlie the emergence of several lineages of eukaryotic organisms as opposed to the idea of all eukaryotes descending from a single universal ancestor (7).

It is important to note that the presence of bacterial HGT signatures in eukaryotic genomes does not depend solely on the ability of bacteria to transfer DNA into the host cell. Instead, four additional major conditions should be met. First, the transferred DNA must become integrated into the host genome. Second, the foreign sequence must not be lost after rearrangements of the genome during subsequent cell divisions. Third, for multicellular eukaryotes, the transformed cell must either be fixed in the germline for genetic modification of animals and plant germlines or regenerate into a viable organism when asexual reproduction is possible, for example, via cell dedifferentiation in plants. Finally, the integrated sequence must be preserved in the course of evolution, which is more likely to occur if the acquired gene confers a selective advantage or is at least neutral rather than deleterious. Thus, transient transformation events, which, by definition, are not retained in the genome, probably occur at a much higher rate than is suggested by the HGT signatures discovered in eukaryote genomes. Yet, this phenomenon of “transient expression,” which is well known in the *Agrobacterium*-plant interaction, might play a role in promoting HGT. For example, hypothetically, transient genetic transformation could express putative effector proteins, which are analogous to effectors translocated from many pathogenic or symbiotic bacteria to their eukaryotic hosts, which in turn will facilitate subsequent rounds of bacterial infection.

The very wide range of eukaryotic cells that can be transformed by *Agrobacterium* suggests that the DNA transfer involves fundamental biological processes common to most, if not all, eukaryotes and is not dependent on host species-specific factors (37). Indeed, in addition to numerous plant species that can be transformed by *Agrobacterium* either naturally or under laboratory conditions, other, evolutionarily distant eukaryotes such as yeast (70, 71) and many other fungi (72, 73) and arachnid (74) and human cultured (75) cells are amenable to *Agrobacterium*-mediated transformation. Moreover, under laboratory conditions, DNA transfer via conjugation-related mechanisms is possible between other bacteria, e.g., *E. coli*, and several eukaryote species. Thus, the combined potential of different bacterial species to modify genetically cells of virtually all eukaryotes supports the notion of HGT as a widespread mechanism in evolution.

## POTENTIAL APPLICATIONS

Besides its importance for understanding the evolution of modern eukaryotes, the bacterium-to-eukaryote gene transfer has a unique and highly significant application potential, which lies mainly in two major areas, research and biotechnology. Research applications mainly aim at discovering new protein functions and cellular pathways by expressing specific genes of interest, delivering gene-targeting systems, such as CRISPR/Cas9 (76), or insertional mutagenesis of genomes of interest, for example, by generating T-DNA insertion mutant libraries of the model plant *Arabidopsis thaliana* (77). Biotechnological applications aim at expressing traits of interest, such as pathogen and abiotic stress resistance genes, genetically engineered pathways for production of biofuels and pharmaceuticals, or generation of desired phenotypes, e.g., color and fragrance of flowers and fruits in agriculture or restoration of normal cellular functions in gene therapy. To achieve these general goals, it is important to adapt and optimize different bacterial DNA transfer systems—or even discover new bacterial species capable of DNA transfer to eukaryotes—for use as vectors for genetic transformation of specific eukaryotic cells or organisms, allowing development of highly efficient “custom-tailored” DNA transfer tools for each specific application.

In plants, *Agrobacterium*-mediated genetic transformation already is efficient for some species, but many other plant species or cultivars, especially those of agronomical importance, are still considered “recalcitrant to transformation” and might become more amenable to gene transfer by a different bacterial vector, for example, belonging to the rhizobial group, with a more appropriate natural host range. Indeed, the early steps of plant genetic transformation rely on close interactions between the bacterium and plant cells, which may be more efficient between plant-associated rhizobia and their hosts than *Agrobacterium* interactions with the same host species. The same approach also may lead to improving the efficiency of transformation of fungal and other eukaryotic species. In fact, the *Agrobacterium*-mediated transformation has become a technique of choice for genetic modification of different species of fungi (73); furthermore, it was also suggested that methods based on bacterium-yeast cell conjugation, using, for example, *E. coli*, could be extended to other species of fungi (78). Obviously, these approaches could be adapted to other eukaryotic cells, such as animal or algal cells.

In animals, two main types of vectors are employed to introduce DNA of interest into human cells for gene therapy: biologicals, such as viruses or bacteria, and biomaterials (79). To date, viruses represent the overwhelmingly predominant vector for use in gene therapy; yet, our increasing knowledge about bacterial DNA transfer systems positions bacteria as a promising alternative for viral vectors (80). Bacterial vectors possess specific features that could be advantageous under specific circumstances: for example, bacterial vectors may be introduced into a tissue for transformation and then easily eliminated by application of antibiotics; many bacteria remain extracellular during DNA transfer, thereby avoiding transfer of DNA sequences other than the gene of interest; and some bacterial strains can target a specific cell type or be engineered for that purpose (80).

Conversely, it is also necessary to investigate and understand potential implications of natural cases of bacterium-to-eukaryote DNA transfer in development of animal and human diseases, such as cancer. Indeed, it is estimated that about 20% of cancers are caused by bacterial or viral infection (81). For instance, one of the best-characterized cases of cancer caused by or associated with bacterial infection is the induction of gastric carcinoma by *Helicobacter pylori* (82). In most cases, it is thought that carcinogenesis results from stress, e.g., tissue inflammation, caused by the infection process (83). However, it has been also proposed that the transfer and insertion of bacterial DNA sequences into the host cell genome may represent another and more specific cause of cancer development (84, 85). If this hypothesis is confirmed, our accumulating knowledge of bacterium-eukaryote DNA transfer will represent an invaluable tool for providing novel insights into early stages of carcinogenesis.

## CONCLUSION

Transfer of genetic information from bacteria to eukaryote cells, once believed to occur exclusively during infection of plants by *Agrobacterium*, probably occurs in many other bacterium-host cell interactions, which include a wide variety of combinations of donor bacterial species and recipient eukaryote species, at least under laboratory conditions. Studies of these gene transfer systems are critical for understanding their potential ecological and evolutionary significance as well as for their utilization for development of new biological tools for fundamental and applied purposes.
